# Human mobility patterns are associated with experienced partisan segregation in US metropolitan areas

**DOI:** 10.1038/s41598-023-36946-z

**Published:** 2023-06-16

**Authors:** Yongjun Zhang, Siwei Cheng, Zhi Li, Wenhao Jiang

**Affiliations:** 1 Department of Sociology and Institute for Advanced Computational Science, Stony Brook University, Stony Brook, USA; 2 Department of Sociology, New York University, New York, NY USA; 3 Center for Applied Social and Economic Research, NYU Shanghai, Shanghai, China

**Keywords:** Socioeconomic scenarios, Computational science, Urban ecology

## Abstract

Partisan sorting in residential environments is an enduring feature of contemporary American politics, but little research has examined partisan segregation individuals experience in activity spaces through their daily activities. Relying on advances in spatial computation and global positioning system data on everyday mobility flows collected from smartphones, we measure experienced partisan segregation in two ways: *place-level partisan segregation* based on the partisan composition of its daily visitors and *community-level experienced partisan segregation* based on the segregation level of places visited by its residents. We find that partisan segregation experienced in places varies across different geographic areas, location types, and time periods. Moreover, partisan segregation is distinct from experienced segregation by race and income. We also find that partisan segregation individuals experience is relatively lower when they visit places beyond their residential areas, but partisan segregation in residential space and activity space is strongly correlated. Residents living in predominantly black, liberal, low-income, non-immigrant, more public transit-dependent, and central city communities tend to experience a higher level of partisan segregation.

## Introduction

Spatial segregation is the linchpin of social stratification in the US. Decades of efforts have been poured into examining residential segregation by race and income^1–4^, but limited research has investigated partisan geographic sorting^5^. In recent years, political pundits, policymakers, and the mass public have expressed concern that ordinary Americans are increasingly disliking and distrusting those from different political parties, and scholars remain puzzled by the relationship between affective polarization and partisan geographic sorting^6–9^. Using large-scale voter registration data, a recent study demonstrates the ubiquitous, substantial partisan sorting in residential environments in the US^9^. Locations with higher population density tend to have a large proportion of Democrats^10^. Yet, residential environments cannot capture all social conditions an individual experiences when they visit places over their daily course of activities^11–14^. It remains unclear whether and how individuals sort on partisanship into different activity spaces.

In this paper, we build on prior work regarding activity space and experienced segregation to examine partisan segregation experienced by individuals in major metropolitan statistical areas (MSAs). Using large-scale GPS data with spatial computation techniques, we estimate a place’s partisan segregation (PPS) by calculating the unevenness of its visitors’ partisan composition as opposed to the ideal integration scenario. We then compute a community’s experienced partisan segregation (EPS) by averaging our segregation measure over the places its residents actually visit during their daily routines. This measure not only captures the segregation in the residential space but also in the activity space away from home.

Our primary foot traffic data are GPS pings collected from smartphone users covering roughly 10% of the US population in 2018–2020 via SafeGraph’s COVID-19 Data Consortium. We obtained the anonymous GPS movement data for over 5 million points of interest (POIs), where visitors can be traced to their home census block group (CBG). Though not a random sample of the US population, this data performs reasonably well in cross-validation analysis and has been widely used by scholars to study important behavioral outcomes such as the spread of COVID-19 and spatial-temporal movement patterns after natural disasters^15,16^. We focus on POIs located in 384 MSAs and exclude census block groups with fewer than 10 POIs in the database. Our analytic sample comprises more than 3 million locations that span across over 100,000 census block groups. To measure the partisan distribution of a place’s visitors, we use the L2 political voter file data to compute each CBG’s share of Democratic, Republican, and non-partisan voters and merge them with the GPS foot traffic data above. We also use data from the American Community Survey to measure other CBG-level characteristics.

We measure experienced partisan segregation in two ways. First, we use *place partisan segregation (PPS)* to capture visitors’ uneven exposure to different political parties in a specific location. PPS exhibits substantial regional variations, being more salient in the Northeastern and Southern cities while less pronounced in West and East coastal cities. We also find that places attached to local residential boundaries such as churches and schools have a higher level of PPS, while places such as stadiums, golf courses, country clubs, zoos, malls, gyms, and museums that connect visitors from diverse communities have a lower level of PPS. PPS is higher for places mainly serving local residents. The time series analysis further shows that PPS is associated with mobility patterns, as it peaked during the early COVID-19 pandemic, which is likely due to partisan differences in compliance with lockdown policies. Moreover, we show that PPS is distinct from place racial and income segregation, despite a small positive association between PPS and racial/income segregation at the MSA and POI levels.

Second, we use experienced partisan segregation (EPS) to capture the degree of partisan segregation that residents of a given community experience through their routine visits to different places. Decomposing EPS into components from visiting places solely in their residential neighborhood and places away from home, we show that EPS is greater when residents only visit places in residential areas. But the EPS in residential neighborhoods and places away from home are strongly correlated. We further show that residents living in predominantly black, lower SES, liberal, non-immigrant, more public transit-dependent, and central city communities have a higher level of EPS.

These findings have both important theoretical and policy implications. First, complementing recent research showing extensive residential partisan segregation in the country^9^, we go beyond the residential space to illustrate partisan sorting in *activity spaces*. We provide first evidence showing that like experienced racial and income segregation^17,18^, experienced partisan segregation in activity spaces remains salient. Some types of activity spaces serve as hubs connecting visitors with diverse partisan backgrounds, while other places exhibit severe partisan segregation, especially places primarily serving local residents. Second, our work speaks to an emerging body of literature comparing segregation in both residential space and activity space^12,13^. Previous studies using survey and GPS data show a strong association between residential segregation and activity space segregation, despite that racial and income segregation is higher in residential areas relative to activity spaces. Our analysis shows similar patterns in terms of partisan segregation. Third, Our work shows that partisan segregation experienced by residents is more salient in communities of color, including Black, Hispanic, and Asian communities, and lower SES neighborhoods. More broadly, our findings of substantial experienced partisan segregation in activity spaces can be seen both as an underlying factor for and as a social consequence of political polarization. Interventions for reducing partisan segregation should consider individuals’ exposure not only in residential environments but also in activity spaces.

## Data

### Safegraph monthly patterns

We obtain large-scale monthly mobility flow via SafeGraph COVID-19 Data Consortium. SafeGraph’s monthly pattern data tracks detailed mobility flow information from origin census block groups (CBGs) to destination places (e.g., restaurants, schools, hospitals, churches) based on GPS pings from millions of anonymous mobile devices. To generate origin-destination mobility flow data, SafeGraph uses a mobile device’s common nighttime (6 p.m.–7 a.m. local time) location over the last 6 week period at the level of Geohash-7 granularity (153 m $\times$ 153 m) to define the home location. SafeGraph then aggregates all devices by home CBGs after applying deferential privacy to device count metrics by adding the Laplacian Noise, a way to anonymize residents at the CBG level. SafeGraph offers the total number of device traffic from the origin CBG to the destination location on the monthly basis since 2018. This allows us to build dynamic directional mobility networks over time within MSAs.

SafeGraph’s mobile device users roughly account for 10% of the entire US population^15^. Using voter roll data from the North Carolina’s 2018 general election, scholars find that SafeGraph mobility data are less likely to capture older and non-White voters^19^. To further validate SafeGraph data, we compare the number of residing devices in CBGs with Census’ total population data. Overall, SafeGraph shows great coverage of CBGs in our sample, but minority, low SES, and Democratic-leaning communities are over-sampled (See Section 1, SI Appendix)^19^.

### L2 political data

To quantify the partisan composition of each CBG, we rely on New York University’s L2 Political Academic Voter File. L2 Political provides detailed information on registered voters’ party affiliation. Following Brown et al.^9^’s work, we recategorize non-Democratic and Republican parties into Democratic-leaning and Republican-Leaning. For those independent and non-partisan voters, we treat them as a separate category (labeled “other”). Then, for each CBG, we compute the share of Democratic- and Republican-leaning voters. Note that party affiliation is only recorded in 30 states plus District of Columbia and the imputation of other 20 states’ party data might introduce some biases. Thus, we also present an alternative approach using voting returns to infer partisanship and further break down the analyses by states with observed or imputed party affiliation data (see Section 1, SI Appendix).

### ACS census block group level data

We compile our CBG level’s demographic and socioeconomic backgrounds from American Community Survey 5-year estimates in 2019 via the R *tidycensus* package.

### Measuring segregation

To quantify the level of partisan segregation in a place individuals experience when they travel to, we compute the place-level partisan segregation. The recently available SafeGraph’s weekly and monthly mobility pattern data contain detailed information on visitor patterns in over 4.3 million places in the US. For each place, SafeGraph provides the location name, industrial classification, date range, number of devices and visitors traveling to this location, their origins of census block groups, device types, and other relevant mobility information from 2018 to 2020. Following Chang et al.^20^ and Moro et al.^18^’s work, we utilize the mobility information from the source CBG to the destination place and quantify the degree of segregation based on the time spent by individuals from different social groups.

To compute mobility-based segregation by partisanship in a place, we need to compute the proportion of the time spent in a period by different partisan groups (our main analysis focuses on 1 year range). Given that SafeGraph only shows the source CBG instead of individual demographic information, we need to infer visitors’ partisan composition based on their home CBGs’ partisan distribution. For each CBG, following the approach described above, we obtain the share of registered voters for Democrats or Democratic-leaning (D), Republicans or Republican-leaning (R), and others (OT). Note that others include independent and those non-partisan voters that cannot be recategorized into Democratic-leaning or Republican-leaning^9^. For a place visited by residents from *N* CBGs, we define the proportion,$\tau _p$, for each group *p* as follows.

1 $$\begin{aligned} \tau _{p=D|R|OT}= \sum _{n=1}^{N}{\theta _p\cdot \frac{v_n}{V}}, \end{aligned}$$

where $\theta$ denotes the proportion of group *p* in a CBG, $v_n$ denotes the *n*th census block group’s total visitors to the place in a given time period, V indicates the total number of visitors from all CBGs in its MSA. Similar to prior work on place-level segregation^18^, we compute PPS based on the following Eq. 2, where $\tau _p$ denotes the proportion of the time spent by group *p*. The factor $\frac{3}{4}$ in the equation normalizes the raw PPS score so that it ranges from 0 to 1.

2 $$\begin{aligned} PPS_{i}= \frac{3}{4}\sum _{p=1}^{3}{|\tau _p- \frac{1}{3}|} \end{aligned}$$

Then, we compute each community’s experienced partisan segregation for residents in a certain community *j* using a weighted average of place-level segregation, where the weights ($\eta$) are proportional to the number of visits to place *i* ($i<=M$) from residents in community *j*.

3 $$\begin{aligned} EPS_{j}= \sum _i^M{PPS_{i}\cdot \eta _i} \end{aligned}$$

### Ethical statement

The Stony Brook University Office of Research Compliance approved the Human Mobility and Segregation in the US project under exemption category 45 CFR 46.104.d.4. Informed consent was waived by the IRB at Stony Brook University. GPS data and voter files data were accessed through third-party data vendors. All data used in this project were at the aggregated level and all methods were performed in accordance with the relevant guidelines and regulations.

## Results

### Place partisan segregation varies across geographic areas

**Figure 1:**
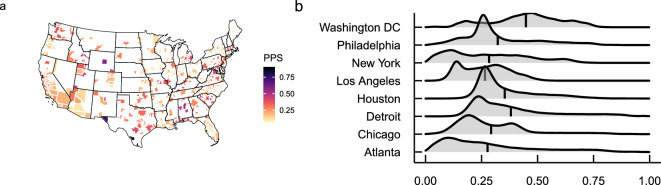
The geographic variation of place-level partisan segregation. The map was produced in R with *tigris* package using the TIGER shapefiles from the US Census Bureau.

Figure 1 maps the geographic variation of PPS. PPS is more salient in the Northeast and South but less pronounced in West and East coastal cities. Overall, the Northeast has the highest average PPS (0.296), followed by the South (0.268), West (0.264), and Midwest (0.258). The national average PPS is 0.264. Figure 1a shows the spatial distributions of PPS. The PPS varies substantially across MSAs, reaching lowest values ($<0.1$) in Bangor (Maine), Ames (Iowa), Burlington (North Carolina), Cedar Rapids (Iowa), Lewiston-Auburn (Maine), Wheeling (West Virginia-Ohio), Des Moines-West Des Moines (Iowa), Dubuque (Iowa), Yuma (Arizona), and Mankato (Minnesota) and highest values ($>0.5$) in Laredo (Texas), McAllen-Edinburg-Mission (Texas), Brownsville-Harlingen (Texas), El Paso (Texas), Daphne-Fairhope-Foley (Alabama), Casper (Wyoming), Dothan (Alabama), Birmingham-Hoover (Alabama), St. George (Utah), and Ann Arbor (Michigan). Figure 1b shows the distributions of PPS at the CBG level for the top 8 MSAs across different regions. It shows substantial variation in PPS within MSAs. The average PPS in Washington DC metro area is 0.448, followed by Detroit (0.381), Houston (0.354), Philadelphia (0.322), Chicago (0.293), New York (0.283), Atlanta (0.277), and Los Angeles (0.265).

### Place partisan segregation varies across different types of locations

**Figure 2:**
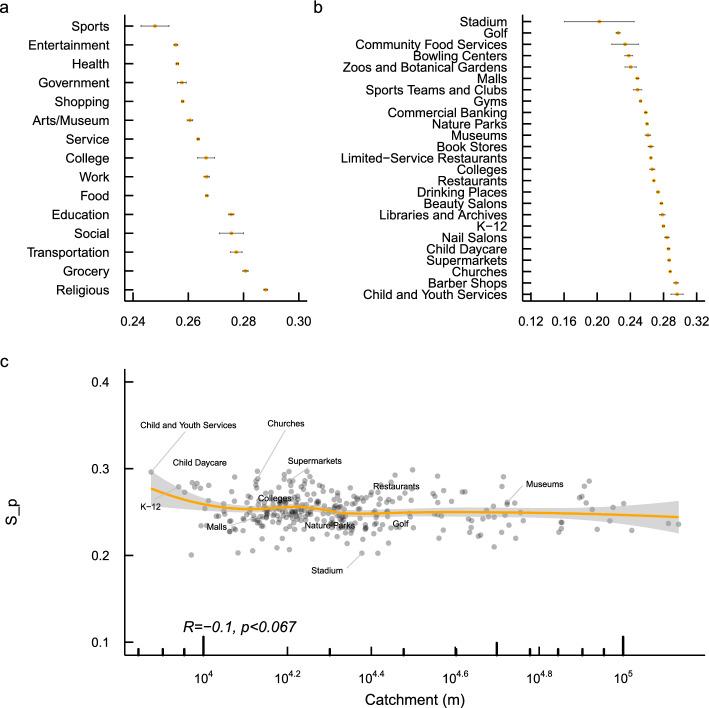
Partisan segregation by location type and catchment range. Error bars indicate 95% confidence intervals.

Figure 2a shows how partisan segregation varies across different types of locations. Following prior work^17,18^, we categorize all places into 15 main categories using the top categories assigned by SafeGraph. Places fostering in-group face-to-face interactions, such as religious organizations and schools, have a higher level of partisan segregation, while places gathering visitors from diverse communities such as sports, entertainment, and shopping have a relatively lower level of partisan segregation. SafeGraph assigns a unique subcategory to each POI, which tracks the detailed type of each place. Figure 2b further shows the distribution of PPS for more detailed categories of theoretical interest. Places such as stadiums, golf courses, country clubs, bowling centers, zoos, malls, gyms, museums, and parks, are integration hubs that gather visitors from politically diverse communities. However, other places, including churches, barber shops, child and youth services, child day care services, and K-12 schools, exhibit a higher level of partisan segregation, partly because these places may be attached to local residential boundaries. In Fig. 2c, we directly examine whether the POI’s catchment size is associated with partisan segregation. We use the average distance of all visitors a POI has to measure its catchment range (in meters). Each dot in this plot represents a subcategory of places (Note that some outliers are removed in this analysis). The plot indicates that catchment is negatively associated with PPS overall (Pearson’s $r=-0.1$, $p<0.067$), but the relationship is highly non-linear, with the bulk of this negative association driven by places within a 10,000 m range. Within this range, if a place serves residents in a larger range, it will host visitors with more diverse partisan backgrounds. However, when the catchment area reaches beyond 10,000 m, catchment area has no effect on a place’s partisan segregation. The POI-level analysis in SI Appendix (Table S4) also shows a similar negative, statistically significant association between catchment and partisan segregation ($\beta =-0.0111$, $p<0.0001$).

### Place partisan segregation increased during COVID-19 lockdown period

**Figure 3:**
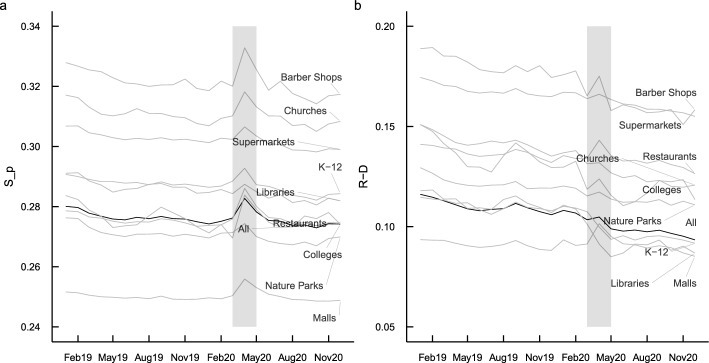
Partisan segregation over time in 2019–2020. The black line denotes all POIs while the gray lines show the other 10 categories of interest.

Figure 3a shows the time series of PPS in 2019–2020. The black line indicates the overall pattern while the gray lines break down the pattern into 10 categories of interest. The lockdown and stay-at-home policies during the early COVID-19 pandemic led to a spike in PPS from March to May 2020 (shaded area) for all place categories. This may be due to partisan differences in compliance with various COVID-19 policies documented by several recent studies^21,22^. To further examine this argument, Fig. 3b shows the difference between the proportion of visitors who are Republicans and the proportion who are Democrats. Consistent with prior findings, this difference exhibits a spike during the lockdown period, suggesting that the spike in PPS observed in Fig. 3a is likely driven by the partisan differences in compliance with lockdown policies.

### Place partisan segregation is distinct from place racial and income segregation

**Figure 4:**
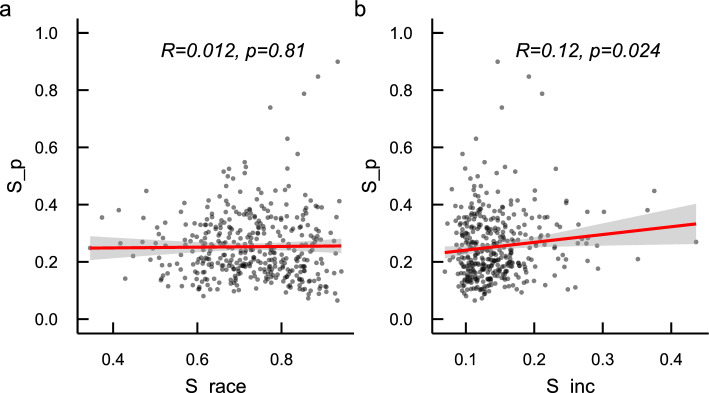
Scatter plots between place-based partisan and racial/income segregation. Each dot is an MSA.

Figure 4 shows the scatter plots between partisan segregation and racial/income segregation at the MSA level. We observe a statistically significant correlation between place partisan and income segregation (Pearson’s $r=0.12$, $p=0.024$) but not between partisan segregation and racial segregation (Pearson’s $r=0.012$, $p=0.81$). Since our segregation measures may depend on the racial, class, and partisan composition in the local area and within the industry, we then regress PPS on racial and income segregation with census tract and North American Industry Classification System (NAICS) fixed effects. The results, shown in Table 1, suggest that the association between place-level partisan segregation and racial and income segregation within a given type of industry and census tract is both positive and statistically significant at the 0.001 level. However, the total within-tract and within-industry variance in partisan segregation explained by income and racial segregation remains rather small (8%), suggesting that place-level partisan segregation captures a distinct dimension of uneven group exposure that goes beyond place-level income and racial segregation.

**Table 1:** Results from fixed-effect models predicting place-level partisan segregation.

	M1	M2	M3
S_race	0.0346***(0.0011)		0.0147***(0.0012)
S_inc		0.0657***(0.0009)	0.0623***(0.0009)
Num.Obs.	3362649	3362649	3362649
R2	0.869	0.869	0.869
R2 Within	0.001	0.007	0.008
BIC	− 9,220,187.6	− 9,240,136.2	− 9,240,952.8
FE: NAICS	X	X	X
FE: Census Tract	X	X	X

*$$p< 0.05$$, **$$p < 0.01$$, ***$$p< 0.001$$.

### Experienced partisan segregation is more salient when residents visit places close to home instead of places away from home

**Figure 5:**
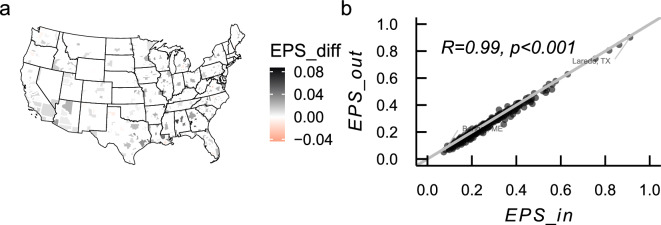
Comparison of EPS within and outside individuals’ home CBG. The map was produced in R with *tigris* package using the TIGER shapefiles from the US Census Bureau.

We next turn to experienced partisan segregation from the community’s perspective. Figure 5 compares EPS within and outside individuals’ home census block groups. We average partisan segregation individuals experience when they visit places in their residential spaces ($EPS\_{in}$) and away from home ($EPS\_{out}$) and compute their difference ($EPS\_{diff}=EPS\_{in}-EPS\_{out}$). Figure 5a shows that the relative EPS difference is positive for most MSAs (88%), but there are 45 (out of 384) MSAs with a negative value, including Ithaca (New York), Charlottesville (Virginia), Bloomington (Indiana), Corvallis (Oregon), the Villages (Florida), etc. Fig. 5b shows the relationship between $EPS\_{in}$ and $EPS\_{out}$. It suggests a strong correlation between partisan segregation experienced within and outside their residential environments.

### Residents living in predominantly black, lower socioeconomic status, liberal, and central city communities tend to experience a higher level of partisan segregation

Table 2 shows ordinary least square regression models predicting CBG-level EPS with controls for MSA-level fixed effects. We use the predominant race group (i.e., the percentage is greater than 0.5) in a CBG as its race (Asian, Black, Hispanic, and White); if there is no predominant race, we label a CBG as Mixed. Results show that compared to residents living in a White community, individuals from predominantly Asian communities experience a lower level of partisan segregation, but other communities of color tend to have a higher level of partisan segregation. We assign a CBG’s partisan label based on their Democratic or Republican share of registered voters, using blue for majority-Democratic CBGs, red for majority-Republican, and purple otherwise. Compared to blue communities, red and purple communities tend to have a lower level of partisan segregation. We use the quartile of median household income, the share of the population with high school or above education, and the share of the population who are employed to measure a CBG’s socioeconomic status. Overall, residents living in lower SES communities experience more partisan segregation. We further assess how immigration, public transit dependence, and the core-suburban status of a community might influence partisan segregation. Following prior work^23,24^, we define whether a community is urban in central cities versus suburban in the corresponding metropolitan area using Census places information. Census places, including both incorporated places (cities, boroughs, towns, and villages) and unincorporated areas, best capture urban, suburban, and rural communities as collectively understood by the residents^24^. We define the central city as the largest place in the metropolitan area by population plus the places with over 200,000 residents, including urban areas such as Jersey City and Newark which have around 248,000 and 277,000 residents in the New York Metropolitan Area. Our results suggest residents living in a community with a lower proportion of the foreign-born population and more public transit-dependent experience more partisan segregation when they visit different locations. In addition, compared to suburban communities, individuals residing in core central cities tend to experience more partisan segregation.

**Table 2:** Results from regression models explaining CBG-level EPS.

	Coefficient	Robust S.E.
Population (ln)	− 0.0009*	(0.0004)
Asian	− 0.0108***	(0.0017)
Black	0.0676***	(0.0009)
Hispanic	0.0209***	(0.0010)
Mixed	0.0051***	(0.0007)
Purple	− 0.0355***	(0.0014)
Red	− 0.0103***	(0.0009)
Income Q2	0.0014*	(0.0006)
Income Q3	− 0.0024***	(0.0007)
Income Q4	− 0.0103***	(0.0008)
% High School or above	− 0.0467***	(0.0026)
% Employment	− 0.0203***	(0.0022)
% Foreign Born	− 0.0464***	(0.0023)
% Public Transit	0.2177***	(0.0025)
Central City	0.0253***	(0.0005)
Num.Obs.	85597	
R2	0.808	
R2 Within	0.369	
BIC	− 241899.3	
FE: MSA	X	

*$$p< 0.05$$, **$$p< 0.01$$, ***$$p< 0.001$$.

### Robustness tests

Our main segregation measure captures how the place-level partisan composition deviates from a reference distribution of evenly distributed partisan groups. In robustness analysis, we consider an alternative reference distribution—the partisan composition at the MSA level. That is, this alternative measure captures how the place-level’s partisan composition deviates from the MSA-level’s. We also report results using voter returns instead of L2 voter registration files to infer visitors’ party affiliation. In addition, we compare our main results with the entropy-based measure. In order to test the sensitivity of our measure to Census geography, we replicate our results using Census tracts instead of CBGs. All robustness test results are available in Section 2, SI Appendix. We anticipate some differences as those alternative measures capture different aspects of partisan segregation but our main results hold across different measures. We also decompose PPS and EPS by CBGs’ features (Sections 3 and 4, SI Appendix) and replicate the main regression results on EPS in Section 5, SI Appendix.

## Discussion and conclusion

In this paper, we systematically examine the extent of partisan segregation experienced by individuals when they move around during their daily activity routines in metropolitan areas. We show that place partisan segregation is more severe in the Southern and Northeastern cities and varies across different types of places. Primary places attached to or serving local residents such as schools and churches tend to have a higher level of partisan segregation, while secondary places such as stadiums, clubs, zoos, and parks tend to have a lower level of partisan segregation. Partisan segregation varies over time and is associated with mobility patterns. For example, there was a peak in partisan segregation during the early pandemic. Moreover, partisan segregation captures a distinct dimension of segregation from racial and income segregation. From the perspective of communities, we show that experienced partisan segregation is higher when a community’s residents visit locations in their residential space than when they visit places away from home, but experienced partisan segregation in both places is strongly correlated. We further show that residents in disadvantaged, liberal, non-immigrant, and central city communities tend to experience a high level of partisan segregation.

These results contribute to the emerging literature in the fields of experienced segregation and activity space. Over the past two decades, Americans appear to be more politically divided than ever^9,25^. Democrats are more likely than Republicans to prefer living in more Democratic, dense, and racially diverse communities^25^, and a large proportion of American voters live with virtually no exposure to outpartisans in their residential environments^9^. But we know little about experienced partisan segregation in individuals’ routine activities. Our study extends partisan geographic sorting in residential environments to sorting in individuals’ activity spaces. Meanwhile, we extend the emerging experienced segregation literature focusing on racial and income segregation to a new political dimension^17,18^. We call for future work to attend to the multidimensional social space of experienced segregation.

Following seminal work by activity space scholars^12,13,26^, our work contributes to the debate on re-conceptualizing neighborhoods as activity spaces instead of focusing solely on residential environments, as residential space cannot capture all social conditions experienced by an individual. On the other hand, our work also shows that partisan segregation in activity spaces and residential environments is highly correlated. The level of experienced partisan segregation may depend on residents’ capacity to travel a long distance to reach more politically diverse communities—indeed, our findings suggest that communities heavily relying on public transit tend to have a higher level of partisan segregation.

Our findings also suggest that to reduce partisan segregation the intervention should pay attention to activity spaces beyond the residential environment. Our results show that public spaces that are not attached to residential boundaries such as stadiums, libraries, zoos, nature parks, and museums can increase individuals’ exposure to outpartisans. Thus, to address the persistent political segregation in the US, policymakers should consider these public infrastructures’ potential for enhancing integration.

The current research has several limitations that readers should be cautious about when interpreting our results. First, because of privacy protection, we lack individual device-level demographic information. CBG-level features are used to infer visitors’ characteristics. This might induce some ecological biases, for example, if individuals who travel more often to places are systematically different from those who mostly stay home. We further conducted simulation studies using ecological inference techniques to examine the visiting patterns for churches and grocery stores. The results indicate that our main arguments still hold, even though our approach might underestimate the level of partisan segregation for places with strong partisan preferences (see Section 6, SI Appendix). Second, showing up in the same place does not necessarily imply social interaction, hence, our experienced segregation measures are better interpreted as capturing potential opportunities for interaction rather than actual interaction. Third, without ruling out unobserved community-level confounders that affect both community characteristics and partisan sorting, our exploration of potential predictors for experienced partisan segregation cannot be interpreted as causal. Finally, due to the lack of data on the precise length of visits in each location, the use of total number of visits in our segregation measures is imperfect. This warrants further research. Despite these limitations, these results point out some important future directions for researchers who study experienced segregation in activity spaces.

## Supplementary Information

Supplementary Information.

## Data Availability

All aggregated data needed to evaluate the conclusions in the paper are shared via an Open Science Framework repository (https://doi.org/10.17605/OSF.IO/K8BES). The original SafeGraph mobility data were not allowed to share publicly due to the licence restriction.
