# Supplementary material for: Human mobility patterns are associated with experienced partisan segregation in US metropolitan areas
Source: Sci Rep. 2023 Jun 16;13:9768. doi: 10.1038/s41598-023-36946-z (PMC10276023; doi:10.1038/s41598-023-36946-z)
Supplement: Supplementary file 1 — Supplementary Information. [file 41598_2023_36946_MOESM1_ESM.pdf]

Supplementary Materials for

Human Mobility Patterns Are Associated with Experienced Partisan Segregation in US Metropolitan Areas

Yongjun Zhang et al.

*Corresponding author. Email: Yongjun.Zhang@stonybrook.edu

This PDF file includes:

- Supplementary Text
- Figs. S1 to S11
- Tables S1 to S5

1 SafeGraph and L2 Data Validation

1.1 SafeGraph Data

SafeGraph data has been widely used by social scientists in mobility-related research. Here we further validates its representativeness. We focus on SafeGraph's number of residing devices at the CBG level in the United States. We first compare it with the total population using data from American Community Survey and then explore potential factors driving the variation in the number of residing devices. Note that we do not have individual demographic information for these devices. Hence, we can only examine whether these residing devices are systematically oversampled in certain communities. But other scholars using vote turnout data from L2 with SafeGraph's traffic data to polling stations show that SafeGraph tend not to capture older and non-White voters[1].

1.1.1 Comparing SafeGraph's Devices Number with Census' Total Population at the CBG Level.

We merge SafeGraph's 2019 home_panel_summary.csv file with the 2019 American Community Survey (ACS). We only keep CBGs with at least one resident in ACS. The Pearson's correlation coefficient between total population estimates and number of residing devices is 0.590. We further restrict CBGs to those resided in metro areas and the correlation coefficient is 0.592. Overall, the number of residing devices at the CBG level from Safegraph is strongly correlated with the total population from ACS.

1.1.2 Predicting Potential Bias in the Number of Residing Devices at the CBG Level in the U.S. Metro Areas.

Given the count nature of the number of residing devices, we first estimate an MSA level fixed-effects Poisson model to explain what factors might account for the potential variation in the number of residing devices at the CBG level. The first column in Table S1 shows potential factors associated with devices. Obviously, the number of residing devices is positively associated with the CBG total population and being non-White (except Asian), Democratic-leaning, lower-income, high employment, or more public-transit usage communities. The second column shows the MSA level fixed-effects OLS model predicting the device-total population rate at the CBG level. The third column shows OLS coefficients predicting the Z-score of device-population rate. Both models suggest similar patterns with the first column.

Table S1. Regression Results Predicting SafeGraph Device Numbers at the CBG Level.

	#Devices	#Devices/Population	#Devices/Population Z-Score
Population (ln)	0.8835***(0.0066)		
Asian	−0.0396(0.0277)	−0.0081(0.0042)	−0.0168(0.0086)
Black	0.1446***(0.0153)	0.0157***(0.0017)	0.0324***(0.0034)
Hispanic	0.1052***(0.0165)	0.0071**(0.0025)	0.0146**(0.0052)
Mixed	0.1082***(0.0154)	0.0091***(0.0020)	0.0188***(0.0041)
Purple	−0.0938***(0.0194)	−0.0046*(0.0022)	−0.0094*(0.0045)
Red	−0.1632***(0.0107)	−0.0110***(0.0012)	−0.0226***(0.0025)
Inc Q2	−0.0919***(0.0099)	−0.0138***(0.0015)	−0.0284***(0.0031)
Inc Q3	−0.1335***(0.0109)	−0.0208***(0.0017)	−0.0430***(0.0035)
Inc Q4	−0.1916***(0.0112)	−0.0279***(0.0020)	−0.0576***(0.0040)
% High School (+)	0.0235(0.0369)	−0.0044(0.0069)	−0.0091(0.0143)
% Employment	0.6205***(0.0642)	0.0713***(0.0078)	0.1470***(0.0160)
% Foreign Born	−0.0233(0.0355)	−0.0089(0.0058)	−0.0183(0.0120)
% Public Transit	0.3925***(0.0452)	0.0402***(0.0064)	0.0828***(0.0133)
Num.Obs.	90 840	90 840	90 840
R2	0.467	0.025	0.025
R2 Within	0.391	0.010	0.010
BIC	4 651 925.2	−104 413.5	26 986.2
FE: MSA	X	X	X

* $p < 0.05$, ** $p < 0.01$, *** $p < 0.001$

1.2 L2 Data

L2 data only recorded party affiliation (PID) in 30 states plus District of Columbia and imputed voters' PID in other 20 states. We further compared L2 data with vote share by breaking down states into two categories: Yes-States that recorded PID and No-States that imputed PID. The results did suggest that states with recorded PID show a more consistent strong correlation between L2 measure and vote share measure. For technical definition on these measures, please see Section 2. But the correlation between L2 measure and vote share measure is also very strong (0.60). We present our results in main text using states with both recorded and imputed PIDs.

Table S2. A List of States Recording Voters' Party Identification.

No.	States	Recorded	FIPS
1	Alaska	Yes	02
2	Arizona	Yes	04
3	California	Yes	06
4	Colorado	Yes	08
5	Connecticut	Yes	09
6	Delaware	Yes	10
7	District of Columbia	Yes	11
8	Florida	Yes	12
9	Idaho	Yes	16
10	Iowa	Yes	19
11	Kansas	Yes	20
12	Kentucky	Yes	21
13	Louisiana	Yes	22
14	Maine	Yes	23
15	Maryland	Yes	24
16	Massachusetts	Yes	25
17	Nebraska	Yes	31
18	Nevada	Yes	32
19	New Hampshire	Yes	33
20	New Jersey	Yes	34
21	New Mexico	Yes	35
22	New York	Yes	36
23	North Carolina	Yes	37
24	Oklahoma	Yes	40
25	Oregon	Yes	41
26	Pennsylvania	Yes	42
27	Rhode Island	Yes	44
28	South Dakota	Yes	46
29	Utah	Yes	49
30	West Virginia	Yes	54
31	Wyoming	Yes	56

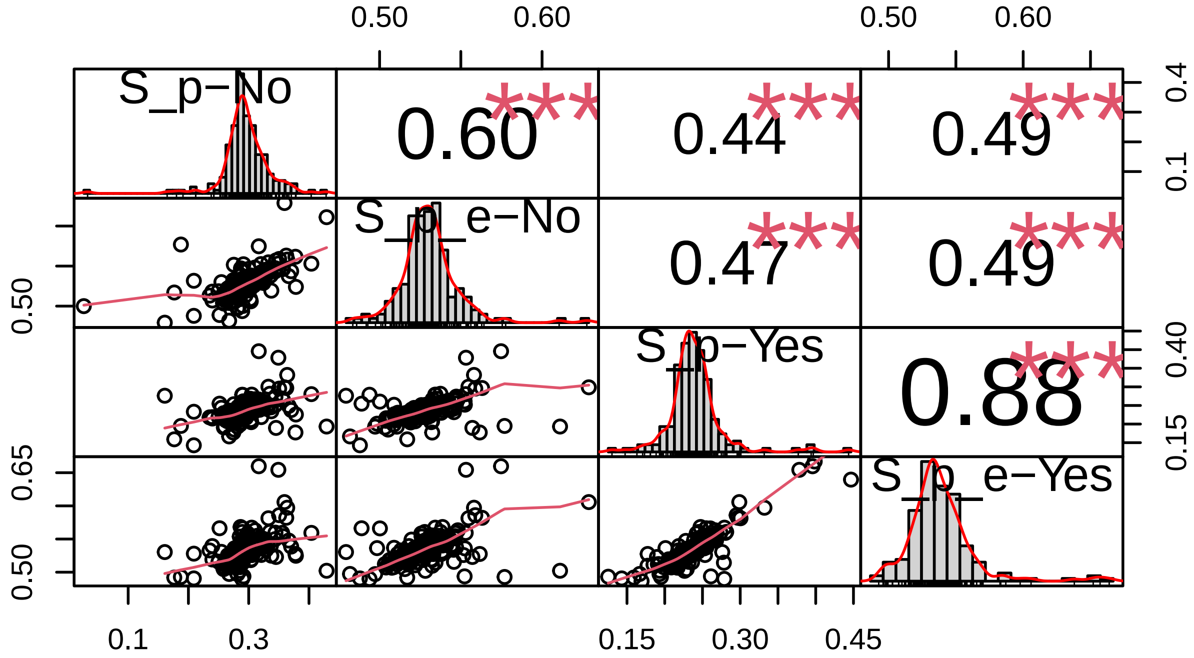

Figure S1. The Correlation Matrix for Partisan Segregation based on L2 and Vote Share. Each dot represents one type of SafeGraph places. Yes and No indicate whether the specific measure was constructed using states that record voters' party affiliation data.

2 Alternative Measures

2.1 Overall Comparison

The place partisan segregation (PPS) measure in our main text, S_p, compares a place's visitors' partisan composition with the ideal integrated scenario (visitors are evenly distributed across different partisan groups). Although this measure captures the relative deviation from the ideal setting, it is sensitive to region-wide partisan composition, as places in a region with a more balanced partisan composition are more likely to be integrated instead of segregated. Thus, we also compute a region-wide adjusted PPS measure, S_p_adj, to capture a place's relative deviation from the MSA-wide pattern. In other words, we compare a place's visitors' composition with the MSA-level partisan composition.

Given that we use L2 voter files to infer individuals' partisan memberships, we also replicate our results using election returns in 2019 to infer the partisanship of visitors from a census block group (CBG). We compute an equivalent measure, S_p_e.

Following prior literature on segregation, we also compute an entropy-based measure, the **diversity ratio**, indicating the deviation of place-level diversity from total diversity in an MSA.

$$Diversity\_Ratio = \frac{E - E_j}{E} \tag{1}$$

The diversity ratio is based on the entropy score, a measure capturing the diversity or evenness of visitors

in a place j, defined as below. Note that m is the group index (Republican, Democratic, and Other), and r_m denotes the proportion of the m group, and E indicates the total entropy in an MSA.

$$E_j = \sum_{m=1}^{M} r_{mj} log(1/r_{mj}) \tag{2}$$

Fig S2 shows the correlation coefficients between our focal measures using L2 data and alternative measures. Note that the entropy score captures the opposite direction of segregation.

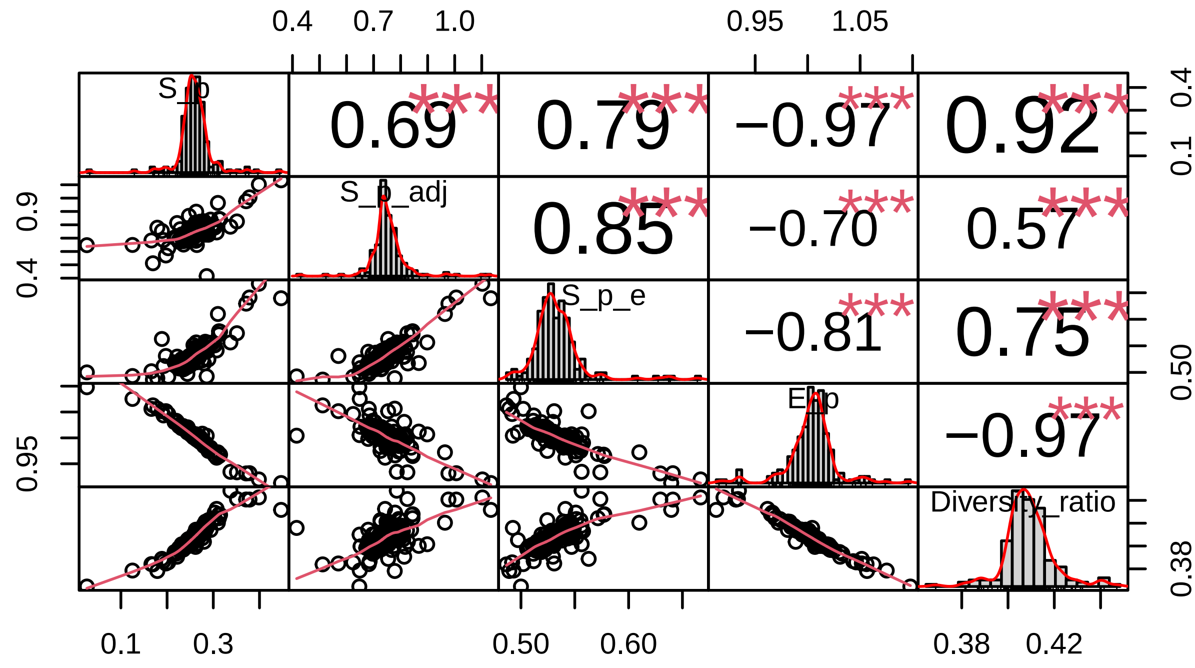

Figure S2. The Correlation Matrix for Alternative Measures. Each dot represents one type of SafeGraph places.

2.2 Mapping Alternative Measures

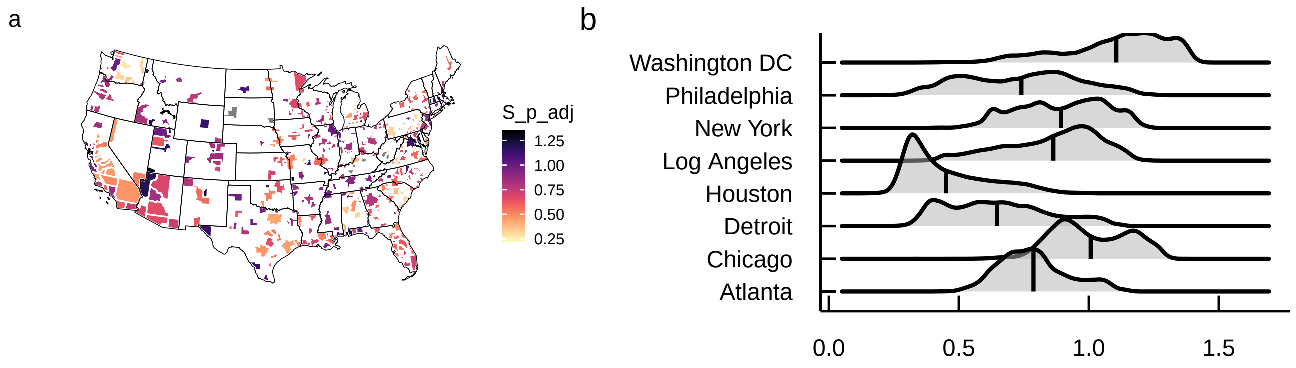

Figure S3. S_p_adj by MSAs. The map was produced in R with *tigris* package using the TIGER shapefiles from the US Census Bureau.

2.3 Replicating the Association of Partisan Segregation with Racial and Income Segregation

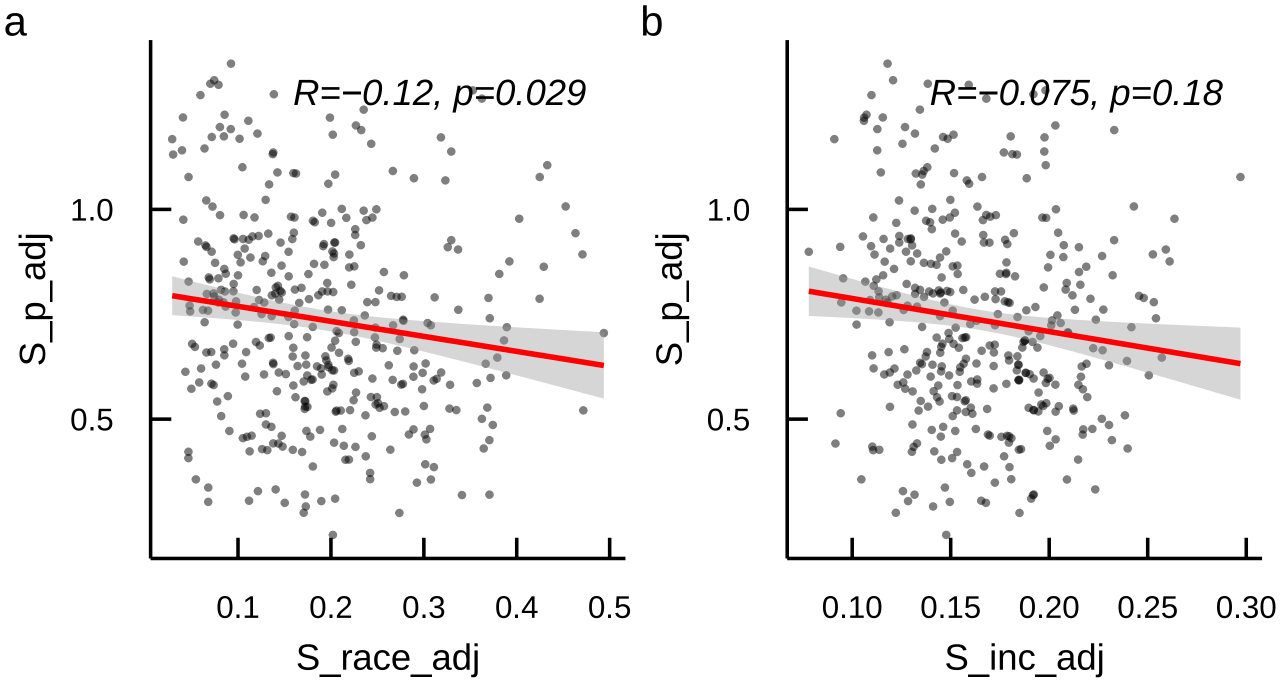

Figure S4. Correlation at the MSA Level.

Table S3. Fixed Effects OLS Results Explaining Alternative PPS Measures at the POI Level.

	S_p	S_p_adj	S_p_e	E_p
S_race	0.015***		0.053***	
	(0.001)		(0.001)	
S_inc	0.062***		0.022***	
	(0.001)		(0.001)	
S_race_adj		0.103***		
		(0.001)		
S_inc_adj		0.014***		
		(0.001)		
E_race				0.035***
				(0.001)
E_inc				0.065***
				(0.001)
Num.Obs.	3362649	3338780	3333834	3358332
R2	0.869	0.946	0.818	0.862
R2 Within	0.008	0.047	0.013	0.024
BIC	−9240952.8	−8482628.8	−12118528.0	−11170558.9
FE: NACIS	X	X	X	X
FE: Tract	X	X	X	X

* $p < 0.05$, ** $p < 0.01$, *** $p < 0.001$

Table S4. Fixed Effects OLS Results Explaining Place-Level Segregation at the POI Level.

	PPS
Total Foot Traffic (ln)	−0.0026***(0.0000)
Catchment (ln)	−0.0111***(0.0001)
Num.Obs.	3088578
R2	0.912
R2 Within	0.024
BIC	−9722594.4
FE: NACIS	X
FE: Tract	X

* $p < 0.05$, ** $p < 0.01$, *** $p < 0.001$

2.4 Replicating Variation in Different Types of Places Using S_p_adj

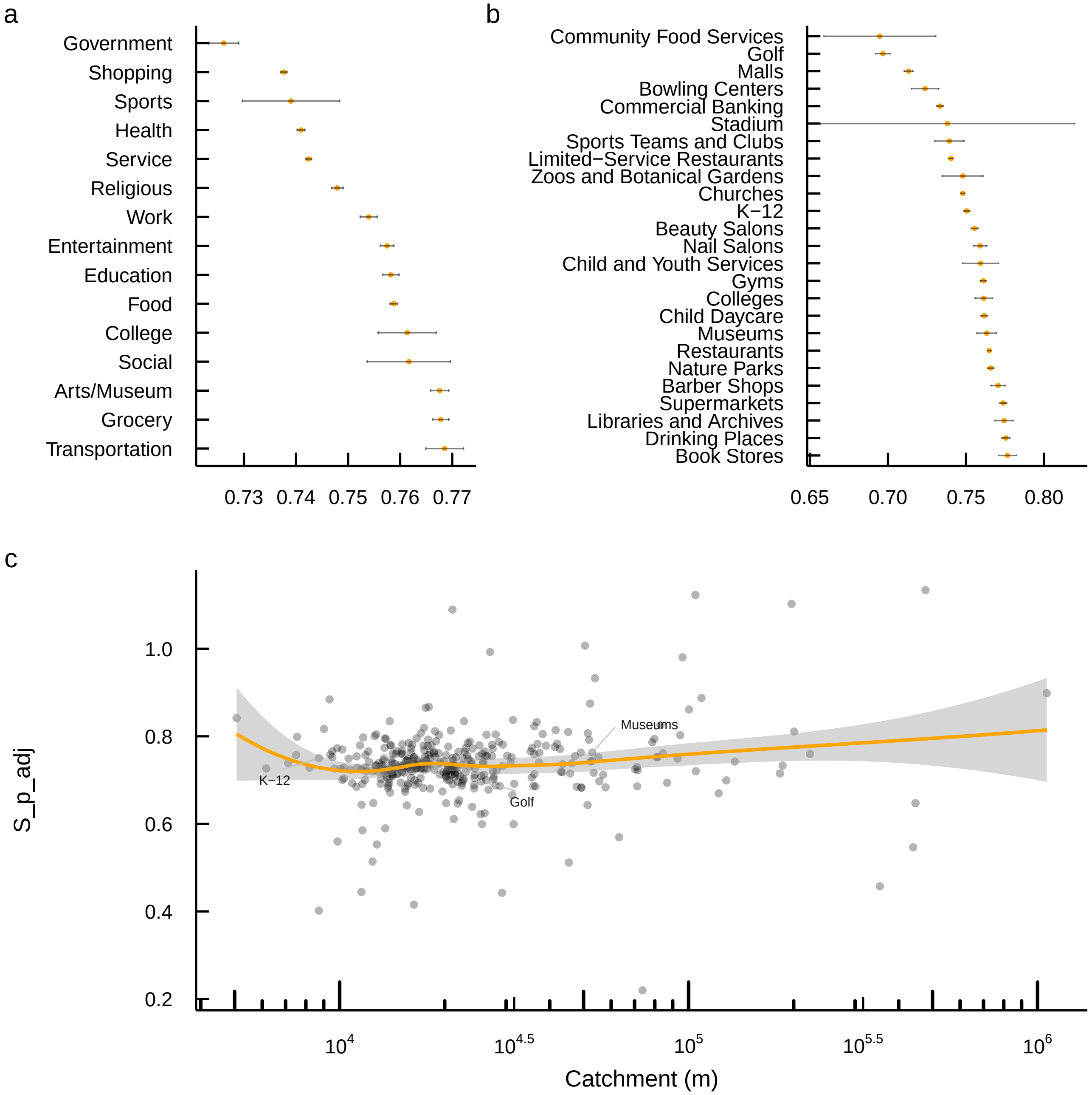

Figure S5. Variation among Different Types of Places.

2.5 Replicating Segregation Measures using Census Tracts instead of CBGs

We also replicate our PPS measure using Census Tract instead of CBG due to the concern of the sensitivity to the choice in Census Geography. Our robustness test shows that the two measures (at tract and CBG levels) are strongly correlated (0.99). Fig S6 compares the distribution of PPS by tract and CBG.

In an ideal setting, our approach to measuring experienced partisan segregation should use individual-level data, but SafeGraph does not provide any demographic information regarding each device. The most

fine-grained data we could obtain is at the CBG-level. To compute community-level EPS, this depends on how scholars define the boundary of a neighborhood. In our case, we use CBG. Since we have all POI's PPS data and associated mobility flow data, it is very flexible to compute EPS at different geographic levels (e.g., Tract and CBG).

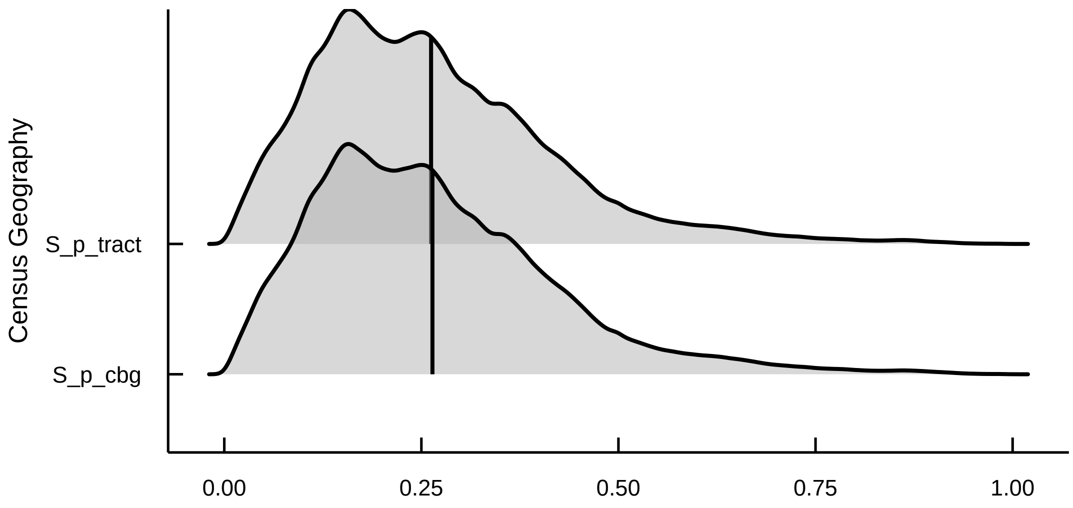

Figure S6. The Distribution of Place Partisan Segregation by Tract and CBG.

3 Decomposing PPS by CBGs' Features

3.0.1 By Race, SES, Ideology, and NAICS.

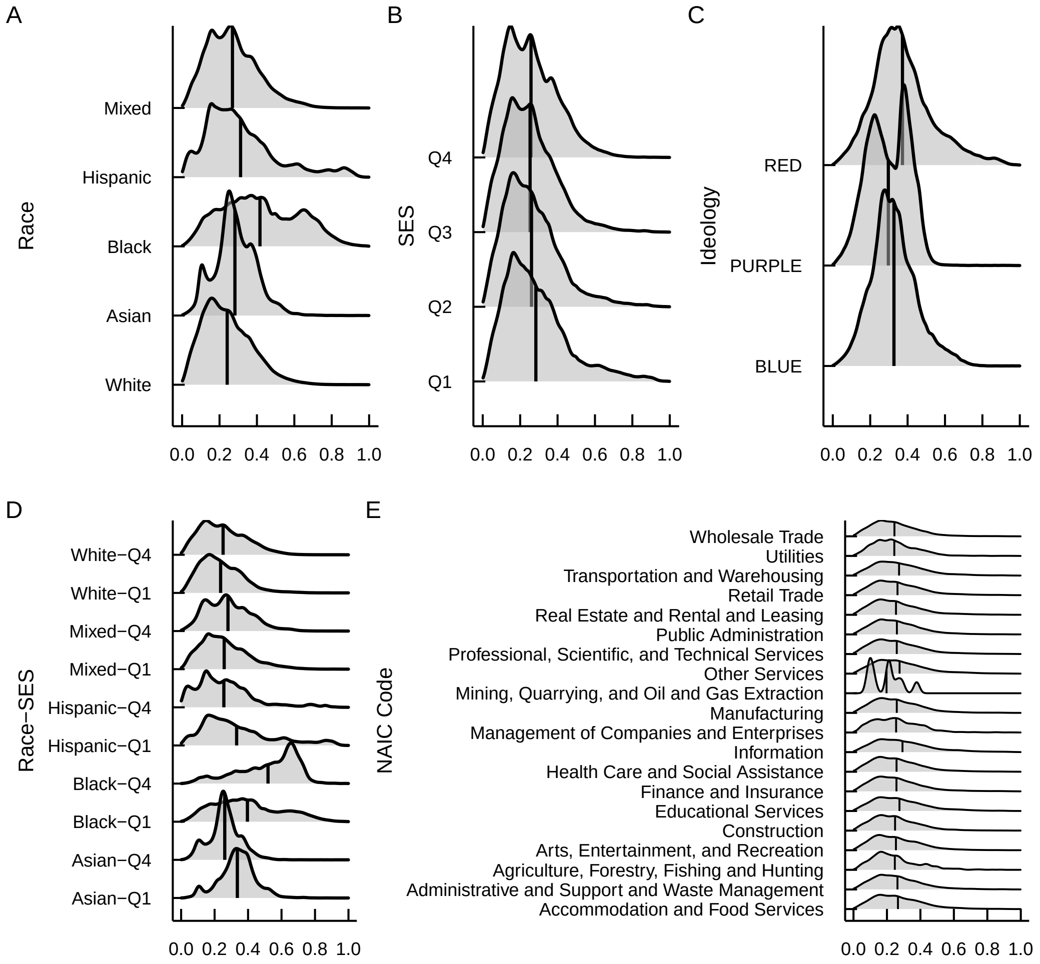

Figure S7. Place Partisan Segregation by Race and SES.

4 Decomposing EPS by CBGs' Features

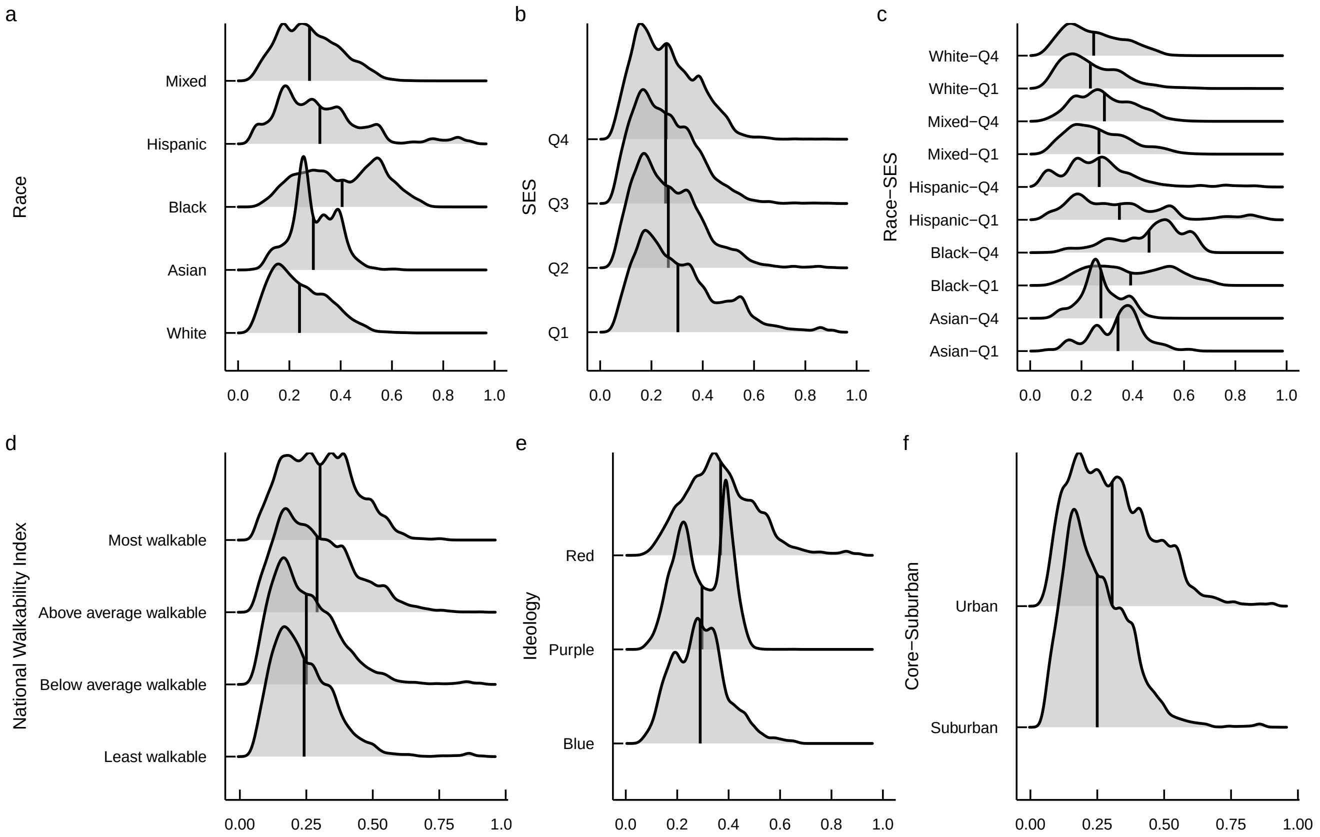

Figure S8. Experienced Partisan Segregation by Race, SES, and Ideology.

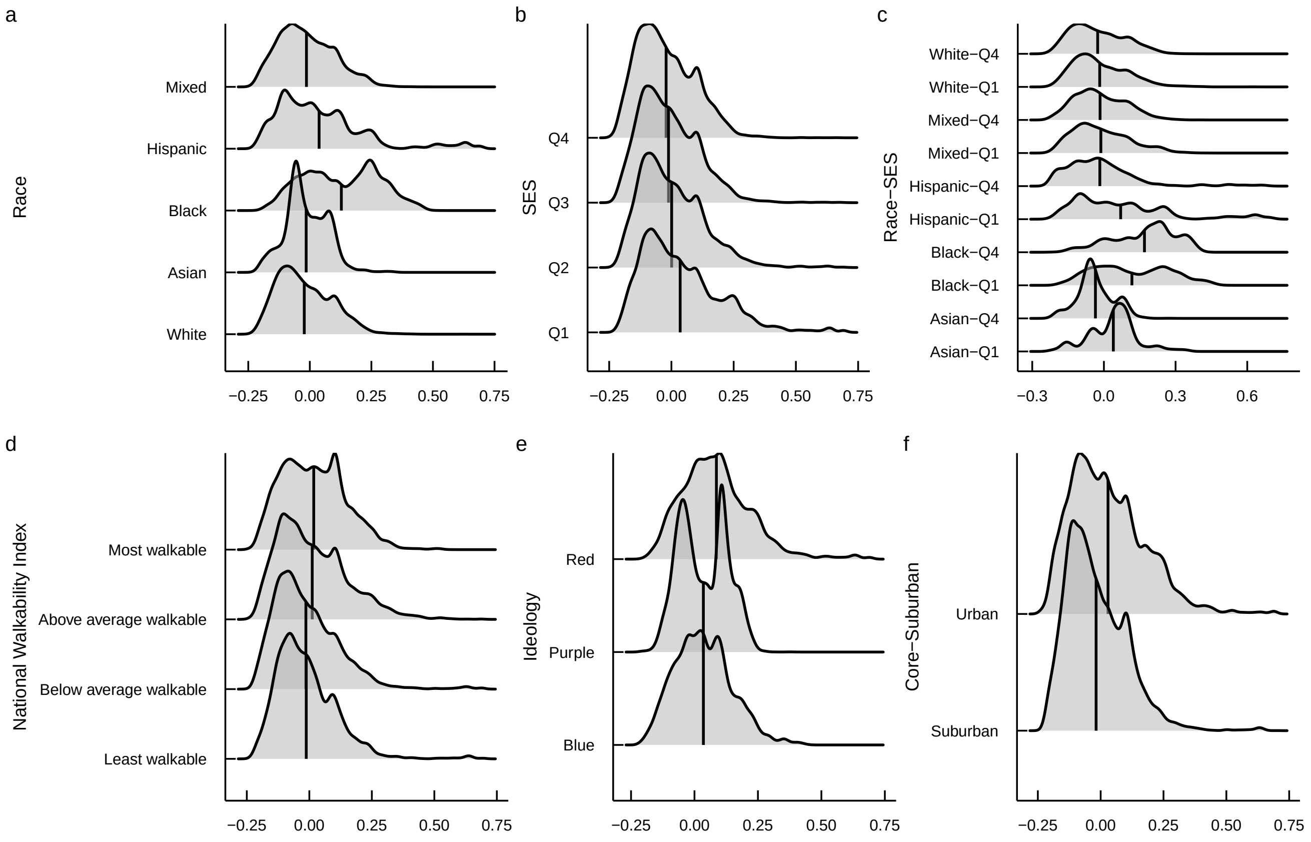

Figure S9. Experienced Partisan Segregation Residuals Net of Racial and Income Segregation by Race, SES, and Ideology.

5 EPS with Alternative Measures

Table S5. Fixed-Effects OLS Results Predicting EPS with Alternative Measures

	S_p_adj	S_p_e
Pop, ln	−0.0004(0.0005)	−0.0027(0.0003)***
Asian	0.0001(0.0018)	−0.0229(0.0015)***
Black	0.0481(0.0010)***	0.0433(0.0006)***
Hispanic	0.0270(0.0012)***	0.0050(0.0007)***
Mixed	0.0066(0.0008)***	−0.0001(0.0005)
Purple	0.0535(0.0019)***	−0.0410(0.0008)***
Red	0.0102(0.0014)***	−0.0136(0.0006)***
Inc Q2	0.0029(0.0008)***	0.0008(0.0004)
Inc Q3	−0.0021(0.0009)*	−0.0043(0.0005)***
Inc Q4	−0.0158(0.0010)***	−0.0097(0.0006)***
High School or Above, pct	−0.0416(0.0032)***	−0.0132(0.0018)***
Employment, pct	0.0013(0.0028)	0.0048(0.0015)**
Foreign Born, pct	−0.0063(0.0026)*	−0.0226(0.0017)***

Public Transit, pct	0.2179(0.0026)***	0.1748(0.0017)***
Central City	0.0280(0.0006)***	0.0156(0.0003)***
Num.Obs.	85 597	85 597
R2	0.935	0.796
R2 Within	0.254	0.374
BIC	−201 200.9	−303 305.3
FE: MSA	X	X

* $p < 0.05$, ** $p < 0.01$, *** $p < 0.001$

6 Issues on Ecological Biases

In this article, we use the partisan composition of a CBG to estimate place partisan segregation based on its visiting patterns. In an ideal setting, we should use individual-level data, but SafeGraph only provides CBG-to-POI level foot traffics. This might result in potential ecological bias. To address the potential ecological bias, we use King's Ecological Inference (EI) that leverages the unequal distribution of within-CBG political affiliations and CBG's outward visitations to different POIs to evaluate the extent to which our estimates are biased[2]. Importantly, the estimates are built upon the assumption that the proportion of visits each POI receives from CBGs is independent of partisan preferences. While other EI options, such as row by columns ($R \times C$) EI are also apt for settings where there are multiple POIs or CBGs (beyond 2 as is often assumed in King's EI), recent simulation studies show that the two methods would produce substantively similar results[3]. We therefore focus on King's EI, using the *R* package *eiCompare*, and report the 95% bound.

Given the computational resources we have, our EI analyses focus on the places in and the visits from New York City, where a number of CBGs are politically heterogeneous. We conduct EI analyses on churches and supermarkets & grocery stores, and expect that individuals from the same CBG have stronger preferences for religious organizations than for purchases of goods for daily uses. We order churches and supermarkets by their total visits in 2018-2020, respectively, and drop the bottom half of the places to reduce measurement error and ensure model computability, as most CBGs have no visits to these places. In total, we include 1500 churches and 1190 supermarkets and grocery stores.

As expected, we observe a larger departure of the EI estimates from our main PPS estimate for churches than for supermarkets in Figure S10, where we present the distribution of the estimates for each location. While our main estimate, the lower-bound, and the upper-bound of EI of PPS for supermarkets are very similar (0.48, 0.51, and 0.53, respectively; see the vertical line of each distribution), the three estimates are more disparate for churches, at 0.50, 0.60, and 0.65. Although we could not extend the analyses to all types of places due to the resource and time restraints, we speculate that we may under-estimate the level of experienced political segregation for places where people from the same CBG have strong partisan preferences (e.g., churches), but not systematically for places where political and ideological preferences are not embedded (e.g., supermarkets and grocery stores). Our substantive results and the ordering of PPS may remain largely robust: places such as churches that we find to have a higher PPS may experience an even greater level of political segregation when ecological biases are accounted for.

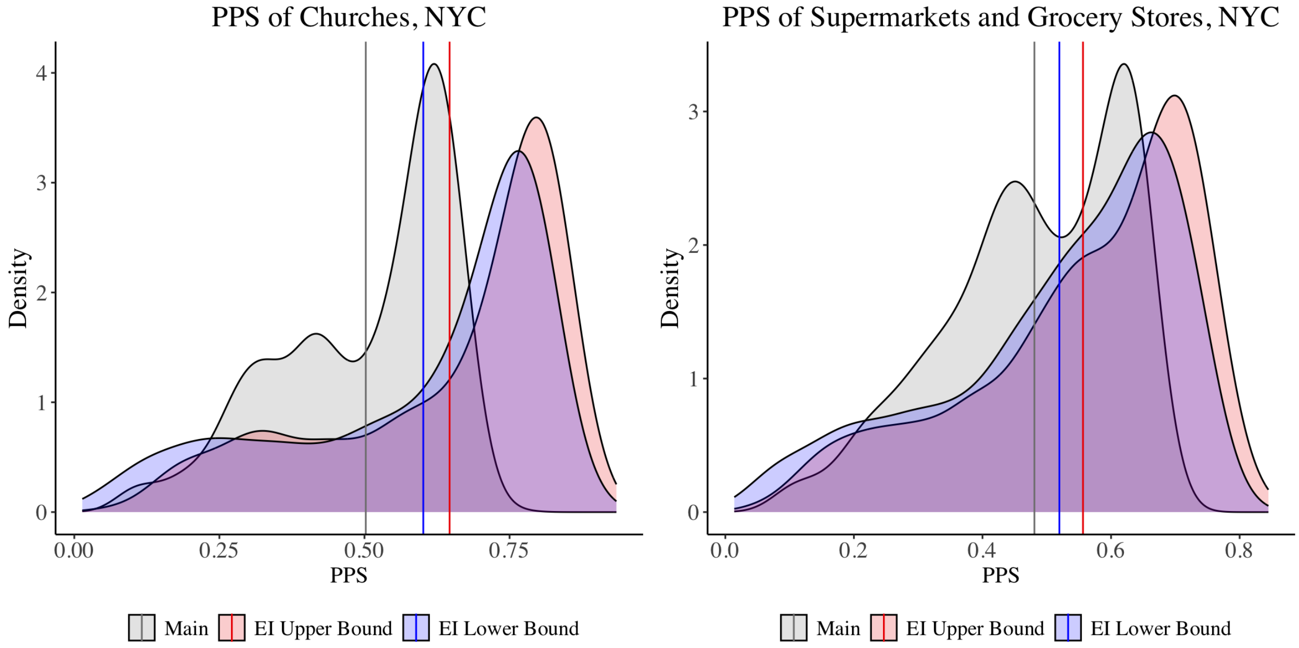

Panel A: PPS of Churches, NYC

Panel B: PPS of Supermarkets, NYC

Figure S10. The Main PPS Estimate and the EI Estimates of Churches and Supermarkets, NYC

To examine whether potential ecological biases pertain to all churches or more to the ones with explicit partisanship such as the ones the reviewer brought up, we classify church's party affiliation by religious denomination in party registration states[4]. Unitarian and Black/AME churches, for example, are explicitly left, while the Evangelist and Brethren churches are majority right. We also switch from NYC to the MSA of Dallas–Fort Worth–Arlington, Texas, where the population is more heterogeneous in political affiliations than the NYC, where the vast majority are Democrats. This can be seen from Figure S11, where our main estimates are significantly lower than the ones in NYC regardless of church's political affiliation. We also find that, while we may underestimate the PPS of churches, the bias is more pronounced for those with explicit political affiliations (whether Democrat or Republican), but not to the ones where partisanship is not explicit. Our main estimate of churches of mixed political affiliation, indeed, is within the 95% bound EI computes. This suggests that biases may appear when people from the same CBG have strong political preferences in the visited place, but not for those where political preferences and connections are weak. Overall, the potential ecological bias does not change our main results, especially regarding the ordering of PPS across different types of places.

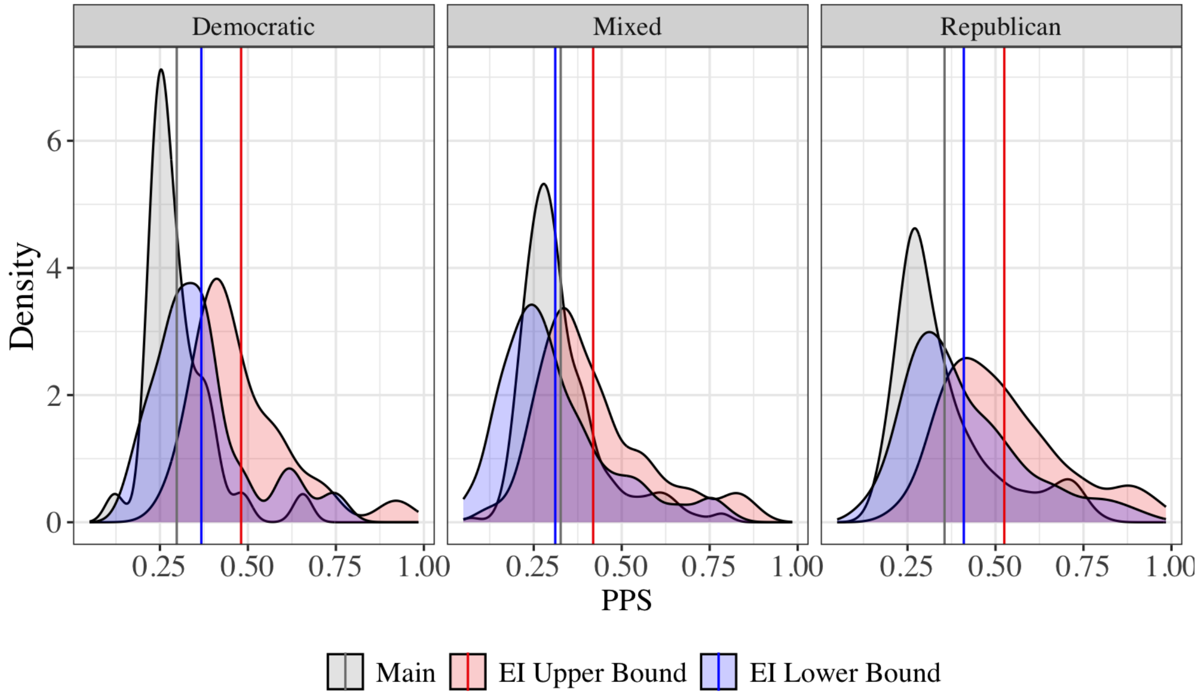

Figure S11. The Main PPS Estimate and the EI Estimates of Churches by Church Partisanship, Dallas–Fort Worth–Arlington MSA, TX

References

1. Coston, A. *et al.* Leveraging administrative data for bias audits: assessing disparate coverage with mobility data for covid-19 policy. In *Proceedings of the 2021 ACM Conference on Fairness, Accountability, and Transparency*, 173–184 (2021).

2. King, G. *A solution to the ecological inference problem: Reconstructing individual behavior from aggregate data* (Princeton University Press, 1997).

3. Barreto, M., Collingwood, L., Garcia-Rios, S. & Oskooii, K. A. Estimating candidate support in voting rights act cases: Comparing iterative ei and ei-r× c methods. *Sociol. Methods & Res.* **51**, 271–304 (2022).

4. Malina, G. & Hersh, E. The politics of 130,000 american religious leaders: A new methodological approach. *J. for Sci. Study Religion* **60**, 709–725 (2021).
